# BgaA acts as an adhesin to mediate attachment of some pneumococcal strains to human epithelial cells

**DOI:** 10.1099/mic.0.045609-0

**Published:** 2011-08

**Authors:** Dominique H. Limoli, Julie A. Sladek, Lindsey A. Fuller, Anirudh K. Singh, Samantha J. King

**Affiliations:** 1 Center for Microbial Pathogenesis, The Research Institute at Nationwide Children’s Hospital, OH 43205, USA; 2 The Ohio State University College of Medicine, Columbus, OH 43210, USA

## Abstract

*Streptococcus pneumoniae* colonization of the respiratory tract is an essential precursor for pneumococcal disease. To colonize efficiently, bacteria must adhere to the epithelial-cell surface. *S. pneumoniae* possesses surface-associated exoglycosidases that are capable of sequentially deglycosylating human glycans. Two exoglycosidases, neuraminidase (NanA) and β-galactosidase (BgaA), have previously been shown to contribute to *S. pneumoniae* adherence to human epithelial cells, as deletion of either of these genes results in reduced adherence. It has been suggested that these enzymes may modulate adherence by cleaving sugars to reveal a receptor on host cells. Pretreatment of epithelial cells with exogenous neuraminidase restores the adherence of a *nanA* mutant, whereas pretreatment with β-galactosidase does not restore the adherence of a *bgaA* mutant. These data suggest that BgaA may not function to reveal a receptor, and implicate an alternative role for BgaA in adherence. Here we demonstrate that β-galactosidase activity is not required for BgaA-mediated adherence. Addition of recombinant BgaA (rBgaA) to adherence assays and pretreatment of epithelial cells with rBgaA both significantly reduced the level of adherence of the parental strain, but not the BgaA mutant. One possible explanation of these data is that BgaA is acting as an adhesin and that rBgaA is binding to the receptor, preventing bacterial binding. A bead-binding assay demonstrated that BgaA can bind directly to human epithelial cells, supporting the hypothesis that BgaA is an adhesin. Preliminary characterization of the epithelial-cell receptor suggests that it is a glycan in the context of a glycosphingolipid. To further establish the relevance of this adherence mechanism, we demonstrated that BgaA-mediated adherence contributed to adherence of a recent clinical isolate to primary human epithelial cells. Together, these data suggest a novel role for BgaA as an adhesin and suggest that this mechanism could contribute to adherence of at least some pneumococcal strains *in vivo*.

## Introduction

*Streptococcus pneumoniae* is a major human pathogen, causing diseases ranging in severity from otitis media and sinusitis to pneumonia, bacteraemia and meningitis (Fine *et al.*, 1996; Leiberman *et al.*, 2001). Pneumococci frequently colonize the nasopharynx asymptomatically (Denno *et al.*, 2002; Watt *et al.*, 2004) and, in the majority of cases, are cleared by the host. Sometimes, however, bacteria can spread to normally sterile sites where they are able to cause disease.

Colonization is an essential step in pneumococcal pathogenesis, but little is known about the mechanisms by which it is established and maintained. Pneumococcal adherence to the epithelial-cell surface is known to play an important role in colonization, and several mechanisms of pneumococcal adherence have already been identified. It is likely, however, that adherence is multifactorial and tissue-specific, and requires additional factors that have yet to be identified (Cundell *et al.*, 1995b; Zhang *et al.*, 2000; reviewed by Hammerschmidt, 2006).

Manipulation of carbohydrates is central to the pneumococcal lifestyle and is a well-utilized mechanism of adherence for both bacterial and viral respiratory pathogens. *S. pneumoniae* has evolved multiple methods to manipulate carbohydrates, including: (i) production of >90 distinct capsular polysaccharides, utilized to protect the organism from opsonophagocytosis (Henrichsen, 1995; Jin *et al.*, 2009; Zartler *et al.*, 2009); (ii) expression of numerous proteins that enable the transport and utilization of carbohydrates (Tettelin *et al.*, 2001); and (iii) production of at least eight surface-associated glycosidases that modify host glycoconjugates (Berry *et al.*, 1994; Bongaerts *et al.*, 2000; Caines *et al.*, 2008; Cámara *et al.*, 1994; Clarke *et al.*, 1995; Jeong *et al.*, 2009; King, 2010; Muramatsu *et al.*, 2001; Umemoto *et al.*, 1977; Zähner & Hakenbeck, 2000).

We demonstrated previously that *S. pneumoniae* can deglycosylate complex *N*-linked glycans sequentially through the activity of neuraminidase (NanA), β-galactosidase (BgaA) and *N-*acetylglucosaminidase (StrH) (King *et al.*, 2006). Neuraminidase NanA is a surface-associated glycosidase that cleaves α2,3- and α2,6-linked terminal sialic acids from many different glycan structures, exposing other carbohydrate residues. Several studies have proposed that cleavage of terminal sialic acid by NanA may reveal a receptor on the epithelial-cell surface. Tong *et al.* (1999) demonstrated increased *S. pneumoniae* binding to neuraminidase-treated chinchilla tracheas, and pneumococcal adherence to human epithelial cells was inhibited by addition of sialylated glycoconjugates (Barthelson *et al.*, 1998). Furthermore, a *nanA* mutant was significantly reduced in adherence to epithelial-cell lines and this deficiency was restored by pretreatment with purified neuraminidase (King *et al.*, 2006).

The ability of *S. pneumoniae* to deglycosylate glycoconjugates sequentially suggests that other glycosidases may contribute to the exposure of receptors by cleaving carbohydrates revealed by NanA. Adherence assays using *bgaA* and *strH* mutants revealed that *bgaA*, but not *strH*, contribute significantly to adherence (King *et al.*, 2006). These data suggest that NanA and BgaA may be deglycosylating a glycoconjugate sequentially to reveal an unknown pneumococcal receptor. Furthermore, as NanA and BgaA, but not StrH, were shown to be required for adherence, the structure of the glycan receptor is most likely distinct from complex *N*-glycan structures displayed on host glycoproteins that were shown previously to be deglycosylated sequentially by these three enzymes (King *et al.*, 2006). Previously published work demonstrating that NanA and BgaA, but not StrH, have increased expression during the initial interaction of *S. pneumoniae* with human epithelial cells supports a role for these two enzymes in the establishment of colonization (Song *et al.*, 2008).

BgaA is a 2228 aa protein that is secreted and attached to the pneumococcal surface in a sortase-dependent manner (Kharat & Tomasz, 2003; Zähner & Hakenbeck, 2000). It is the only β-galactosidase expressed by *S. pneumoniae* with sequence similarity to *Escherichia coli* LacZ, although it is over twice the size of this enzyme and the vast majority of other bacterial β-galactosidases (1024 aa). This larger size may suggest a secondary function for the protein. Pneumococcal BgaA has high specificity for terminal galactose β1,4-linked to *N-*acetylglucosamine (Galβ1,4GlcNAc), indicating that, unlike other characterized β-galactosidases, the main role for BgaA is not in lactose metabolism, but instead in deglycosylation of human glycoconjugates (Zähner & Hakenbeck, 2000; Zeleny *et al.*, 1997). The gene encoding BgaA is present in all available genome sequences and antibodies to BgaA are found in post-immune serum, suggesting that BgaA is expressed *in vivo* during pneumococcal colonization or disease (Giefing *et al.*, 2008; Hoskins *et al.*, 2001; Lanie *et al.*, 2007; Tettelin *et al.*, 2001; Zysk *et al.*, 2000).

In the present study, we demonstrate that the role of BgaA in adherence is independent of its enzymic activity, and present strong evidence that BgaA acts as a pneumococcal adhesin. Furthermore, we demonstrate that this mechanism is relevant to the adherence of recent clinical isolates to primary human epithelial cells.

## Methods

### 

#### Bacterial strains, culture media and chemicals.

Bacterial strains used in this study are described in Table 1. C06_18 is a low-passage strain isolated at Nationwide Children’s Hospital during 2005–2006. *S. pneumoniae* was grown on tryptic soy (Becton Dickinson) plates with 1.5 % agar, onto which 5000 U catalase (Worthington Biochemical) was spread and incubated at 37 °C in 5 % CO_2_. Pneumococcal broth cultures were grown in Todd–Hewitt broth supplemented with 0.2 % yeast extract (both from Becton Dickinson) (THY). Where appropriate, medium was supplemented with streptomycin (200 µg ml^−1^), kanamycin (500 µg ml^−1^) or erythromycin (1 µg ml^−1^). *E. coli* strains TOP10 (Invitrogen Life Technologies) and XL1-Blue (Stratagene) were grown and maintained in Luria–Bertani (LB) medium. When required, LB was supplemented with ampicillin (100 µl ml^−1^), X-Gal (80 µg ml^−1^) and IPTG (20 mM). All other chemicals, substrates and enzymes, unless otherwise specified, were purchased from Sigma.

**Table 1.** Strains used in this study Cm^r^, Resistant to chloramphenicol; Em^r^, resistant to erythromycin; Sm^r^, resistant to streptomycin, Tet^r^, resistant to tetracycline; UN, unencapsulated.

Strain name	Serotype	Characteristics/genotype	Source or reference
***Streptococcus pneumoniae***			
D39	2	Clinical isolate	Iannelli *et al.* (1999)
R6	UN	Unencapsulated varient of D39	Iannelli *et al.* (1999)
D39Δ*bgaA*	2	Δ*bgaA* (Em^r^)	King *et al.* (2006)
R6Δ*bgaA*	UN	Δ*bgaA* (Em^r^)	King *et al.* (2006)
R6Δ*bgaA/bgaA*^+^	UN	Δ*bgaA/bgaA*^+^	This study
R6Δ*nanA*	UN	Δ*nanA* (Cm^r^)	King *et al.* (2006)
R6Δ*nanA*Δ*bgaA*	UN	Δ*nanA* (Cm^r^), Δ*bgaA* (Em^r^)	This study
R6Δ*cbpA*	UN	Δ*cbpA* (Em^r^)	Dr M. Bender
R6Sm^r^	UN	*rpsL*K56T (Sm^r^)	This study
R6Sm^r^Δ*bgaA*	UN	Δ*bgaA* (Em^r^), *rpsL*K56T (Sm^r^)	This study
R6BgaAE564Q	UN	Gln564→Glu in BgaA, *rpsL*K56T (Sm^r^)	This study
C06_18	22F	Clinical isolate from blood	Burnaugh *et al.* (2008)
C06_18Δ*bgaA*	22F	Δ*bgaA* (Em^r^)	This study
TIGR4	4	Clinical isolate	Tettelin *et al.* (2001)
TIGR4Δ*bgaA*	4	Δ*bgaA* (Em^r^)	This study
384	6A	Transparent variant of clinical isolate P303	Kim & Weiser (1998)
***Escherichia coli***			
One Shot TOP10 chemically competent cells		F^−^ *mcrA* Δ(*mrr-hsdRMS*-*mcrBC*) ϕ80*lacZ*ΔM15 Δ*lacX74* *recA1* *araD139* Δ(*ara-leu*)*7697 galU galK rpsL* (Sm^r^) *endA1* *nupG*	Invitrogen
XL1-Blue supercompetent cells		*recA1 endA1 gyr96 thi-1 hsdR17 supE44 relA1 lac* [F′ *proAB lacl*^q^*Z*Δ*M15* Tn*10* (Tet^r^)]	Stratagene

#### Mutation of *nanA* and *bgaA*.

Insertion–deletion mutations were generated as described previously and confirmed by PCR using primers flanking the constructs (primers: *nanA*, N.1 and N.2, N.3 and N.4; *bgaA*, B.1 and B.2) (Table 2) (King *et al.*, 2004, 2006). All genomic DNA was prepared as described previously (Whatmore *et al.*, 1999). The gene encoding NanA is predicted to be in a single gene transcriptional unit and BgaA is predicted to be the last gene in an operon, therefore polar effects are probably irrelevant (Kaufman & Yother, 2007; Tettelin *et al.*, 2001). The *bgaA* mutant was genetically reconstituted by reintroducing the deleted region into the same genetic location by transformation. Replica plating of resulting colonies was performed to identify erythromycin-sensitive transformants. The presence of the gene was established by PCR and a β-galactosidase activity was used to confirm that functional protein was expressed.

**Table 2.** Primers used in this study

Group/primer no.	Primer sequence (5′→3′)*	Location (GenBank accession no.)
***nanA***		
N.1	TATCGAGTAGGGTAGTTCTT	149–169 (X72967)
N.2	ACGGGGCAGGTTAGTGACAT	1953–1972 (V01277)
N.3	TAGTTCAACAAAGGAAAATTGGATAA	1101–1126 (V01227)
N.4	AGCACGAACTGGAATCTTACCT	1008–1037 (U43526)
**Janus**		
J.1	CCGTTTGATTTTTAATGGATAATG	7–30 (AY334019)
J.2	GGGCCCCTTTCCTTATGCTT	247 511–247 527 (AE005672)
**Plasmid**		
P.1	GTAAAACGACGGCCAG	391–406†
P.2	CAGGAAACAGCTATGAC	205–221†
***bgaA***		
B.1	AACTAGGTTGTCATACCATG	149–168 (AE007374)
B.2	GTAACTACTAATCCTGCACT	6888–6907 (AE007374)
B.3	ATTGTTCGCCTTACTTTTGATG	683–703 (AF282987)
B.4	ACGGGAGTTTGATTTTGGTTGTG	2161–2183 (AF282987)
B.5	TTCATGTGGTCAATTGGTAAT**C**AAATAGGTGAAGCTAATGGTG	1669–1711 (AF282987)
B.6	CACCATTAGCTTCACCTATTT**G**ATTACCAATTGACCACATGAA	1669–1711 (AF282987)
B.7	TAACGGTAACCAAACAAATCCTTC	1254–1277 (AF282987)
B.8	TAGATGCGATTTTAGAAGTTG	1139–1159 (AF282987)
B.9	TGGCGTCTACATGGATTCTCAAGT	702–725 (AF282987)
B.10	CATTATCCATTAAAGCAAATCAAACGGAATCAATTCGTACAAGGCA*^a^*	1197–1215 (AF282987)
B.11	AAGCATAAGGAAAGGGGCCCGGATGTTGATAAGACTCGCTATG*^b^*	1758–1780 (AF282987)
B.12	TTATGCGGATGAAACACTTAT	129–149 (AF282987)
B.13	ACGGGAGTTTGATTTTGGTTGTG	2161–2183 (AF282987)

* Underlining indicates overhang for splicing by overlap extension to primers J.1*^a^* and J.2*^b^*; bold indicates base change for site-directed mutagenesis.

† Location is relative to the described 2.1-TOPO sequence (Invitrogen).

To generate an active-site mutant in BgaA, we performed site-directed mutagenesis of nucleotide 1690 (G→C), which resulted in replacement of glutamic acid-564 with a glutamine residue. A region of *bgaA* flanking glutamic acid-564 was amplified from chromosomal DNA of strain R6 using primers B.3 and B.4, cloned into pCR2.1-TOPO and maintained in TOP10 cells. The presence of the insert was confirmed by PCR (primers P.1 and P.2). In order to minimize PCR-generated errors, all PCRs for mutant generation were conducted using a high-fidelity proofreading polymerase (*Pfx50*; Invitrogen). Plasmid from the resulting strain was prepared (Qiagen) and used as the template for site-directed mutagenesis. The point mutation was introduced using primers B.5 and B.6 and a QuikChange II site-directed mutagenesis kit (Stratagene). Following the mutagenesis, DNA was transformed into XL1-Blue supercompetent cells. The presence of the point mutation and absence of other sequence changes were confirmed by sequencing (primers B.3, B.4, B.7, B.8, P.1 and P.2). To introduce the site-directed mutation into R6, we utilized the Janus cassette selection system (Sung *et al.*, 2001). DNA fragments flanking the mutagenesis site were amplified (primers B.9 and B.10, and B.11 and B.4) and joined sequentially to the Janus cassette PCR product (primers J.1 and J.2) using a variation on the splicing by overlap extension by PCR process (Burnaugh *et al.*, 2008), first described by Horton *et al.* (1989). The Janus construct was transformed into *S. pneumoniae*; transformants were selected on kanamycin and confirmed by PCR (B.12 and B.13). These Janus mutants were then transformed with the PCR product containing the site-directed mutation. Generation of a strain containing the desired mutation was confirmed by PCR with primers flanking the construct (B.12 and B.13) and sequencing. None of the mutants generated showed a significant reduction in growth rate or maximum OD_600_ relative to the parent strain (data not shown). As opacity can also affect adherence of *S. pneumoniae*, all mutants were confirmed as being of the same opacity as their parental strains (Cundell *et al.*, 1995c; Gould & Weiser, 2002; Kim & Weiser, 1998).

#### Adherence of *S. pneumoniae* to human epithelial cells.

The adherence of *S. pneumoniae* to Detroit 562 cells (D562; ATCC CCL-138), a human pharyngeal carcinoma cell line; A549 cells (ATCC CCL-185), a human lung carcinoma cell line; 16HBE14, an immortalized human bronchial epithelial cell line (Gruenert *et al.*, 1995); LA-4 cells (ATCC CCL-196), a mouse lung adenoma epithelial cell line; and primary normal human bronchial epithelial (NHBE) cells, was determined essentially as described previously (Gould & Weiser, 2002; King *et al.*, 2006). In brief, bacteria were grown in liquid culture to an OD_600_ of 0.6, diluted in antibiotic-free tissue-culture medium and, on average, 5×10^4^ bacteria per 1.9 cm^2^ were added to a confluent monolayer of epithelial cells. The bacteria were applied to the epithelial cells by centrifugation (250 ***g*** for 5 min) and then allowed to adhere at 37 °C for 60 min (no difference in adherence was observed in control experiments in the absence of centrifugation). To determine the percentage of the bacteria from the inoculum that were adherent, non-adherent bacteria were removed by five washes in PBS, and epithelial cells and adherent bacteria were lifted with 0.25 % trypsin/1 mM EDTA, vortexed and maintained at 4 °C. Bacteria were enumerated by serial dilution. The percentage adherence of all strains was calculated as the proportion of the inoculum that was adherent to the epithelial cells. Adherence of the wild-type strains was adjusted to 100 % and adherence of the mutants was expressed relative to that of the parental strain under the same experimental conditions. All adherence assays were performed in triplicate on at least three independent occasions. Data are presented as means±sd of the independent experiments.

This method was modified for NHBE cells, which were washed three times instead of five after incubation with pneumococcal strains to prevent lifting of epithelial cells. Means of at least three independent experiments were used for statistical analysis. Where indicated, D562 cells were pretreated for 30 min with 0.01 U purified *Clostridium perfringens* neuraminidase per 1.9 cm^2^ well surface area. *C. perfringens* neuraminidase has cleavage specificities the same as those of the pneumococcal neuraminidase, NanA (Cassidy *et al.*, 1965). Assays in which the adherence of R6 was blocked by recombinant BgaA (rBgaA) (QA Bio) were performed by adding 4.33 pmol (unless otherwise indicated) rBgaA per 1.9 cm^2^ well surface area with bacterial inoculum to neuraminidase-treated cells. Dose dependence was evaluated by incubating D562 cells in concentrations of rBgaA ranging from 0.00433 to 4.33 pmol per 1.9 cm^2^ well surface area. Trypan blue staining was used to confirm that purified neuraminidase and rBgaA were not toxic to epithelial cells. To perform staining, cells were treated with glycosidases, washed and lifted with trypsin as described above. Subsequently, 25 µl cells was added to an equal volume of 0.4 % trypan blue. Following incubation at room temperature for 2–3 min, total numbers of cells and stained cells were counted by using a haemocytometer.

To determine the role of G-protein-coupled platelet-aggregating factor receptor (PAFR) in adherence of R6 in our system, we added 1 or 10 µM PAFR antagonist to the bacterial inoculum. The contribution of proteinaceous receptors to pneumococcal adherence was tested by preincubating D562 cells with 0.0025 % trypsin or 1 mg proteinase K ml^−1^ for 10 min at 37 °C. Following treatment, cells were washed twice with PBS and adherence assays were performed as described above. To investigate the role of asialo-GM1, adherence to neuraminidase-treated D562 cells was determined in the presence of a range of concentrations of rabbit anti asialo-GM1 (Wako Chemicals) or asialo-GM1.

#### Labelling of carboxylate-modified FluoSpheres with rBgaA.

rBgaA, glycine (Acros Organics) or BSA were bound covalently to carboxylate-modified FluoSpheres (Molecular Probes) (505/515 nm range, yellow, 1 µm in diameter) as described previously (Romero-Steiner *et al.*, 2006). In brief, the negative charge of the sonicated, washed, carboxylate-modified FluoSpheres was activated by EDAC solution (100 mg ml^−1^) (Molecular Probes). A 250 µl volume of rBgaA (1 mg ml^−1^), BSA (0.5 mg ml^−1^) or glycine (1 M) was added to the FluoSpheres and incubated overnight at room temperature. The remaining active sites were blocked by addition of a 1 M solution of glycine, followed by a 30 min incubation. The FluoSphere suspension was then washed three times in PBS and the supernatant of the first wash was saved for protein determinations using a Micro BCA Protein Assay kit (Pierce).

#### Adherence of rBgaA-coated FluoSpheres to D562 cells.

D562 cells were grown to confluence in 24-well tissue-culture plates and were pretreated for 30 min with 0.01 U purified *C. perfringens* neuraminidase per 1.9 cm^2^ well surface area. Non-treated cells were incubated with medium alone. The cells were washed once with 0.5 ml PBS buffer+0.5 % BSA per well. Preliminary experiments identified a 1/250 dilution of FluoSpheres in PBS buffer+1 % BSA as optimal for measuring adherence. A 200 µl volume of a sonicated (3 min), diluted FluoSphere suspension or a no-FluoSphere control was added to the centre of the washed monolayers and assays were performed as described previously (Romero-Steiner *et al.*, 2006). In brief, the beads were allowed to adhere for 2 h at 37 °C (5 % CO_2_), the plates were then washed five times with PBS+0.5 % BSA and allowed to dry, and adherence was assessed using a fluorometer (485/520 nm). Experiments were performed in triplicate on three independent occasions.

#### β-Galactosidase activity assays.

β-Galactosidase assays were performed using a modification of the colorimetric assay using ONPG as a substrate, as described previously (King *et al.*, 2006). Assays were performed in triplicate on three independent occasions.

#### Mouse model of pneumococcal colonization.

Animal experiments and procedures were conducted according to protocols approved by the Research Institute at Nationwide Children’s Hospital Institutional Animal Care and Use Committee. Nasopharyngeal colonization was performed essentially as described previously (McCool & Weiser, 2004). For C06_18 and C06_18Δ*bgaA*, 6- to 8-week-old C57BL/6 mice (Jackson Laboratories) were inoculated intranasally with approximately 2×10^7^ mid-exponential-phase organisms. The density of colonization was assessed by upper respiratory tract lavage and quantitative culture for four mice at 36 h. The animal data are presented as means+sem (c.f.u. ml^−1^).

#### Statistical analysis.

β-Galactosidase activity assays and adherence assays were analysed by two-tailed Student’s *t-*tests.

## Results

### BgaA-mediated adherence is not complemented by exogenous enzyme

**Fig. 1.**
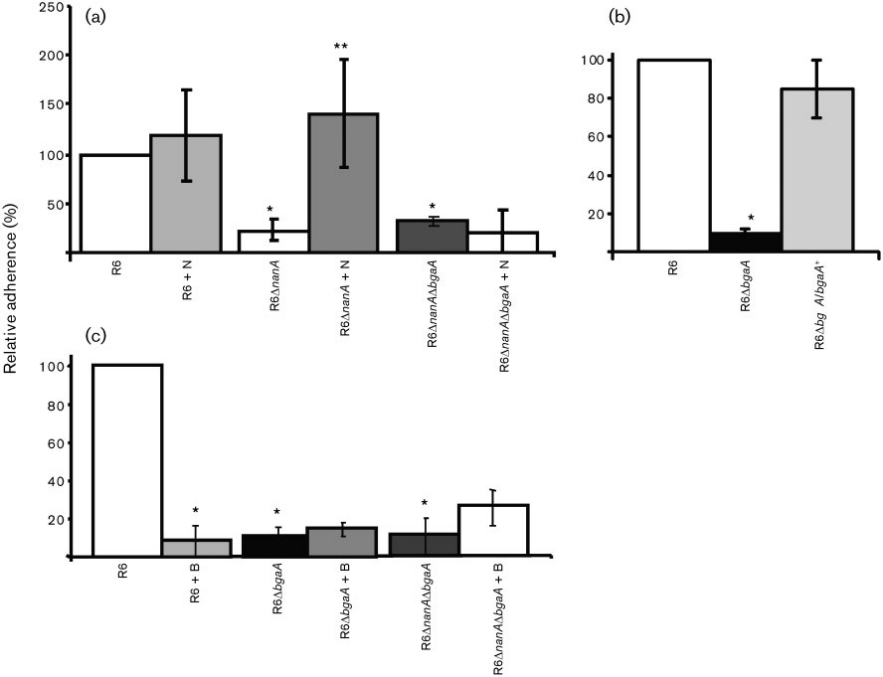
Determination of whether glycosidases can complement the adherence of pneumococcal glycosidase mutants. R6 and R6 exoglycosidase mutants were grown to an OD_600_ of 0.6 and their relative adherence to D562 cells was determined. Adherence is expressed in comparison to the percentage adherence of parental strain R6 over a 60 min incubation period under the same conditions. Values are means±sd of three independent experiments. (a) Purified neuraminidase complements the adherence of a *nanA* mutant, but not a *nanA bgaA* double mutant, to human epithelial cells in culture. N indicates that D562 cells were pretreated for 30 min with 0.01 U purified *C. perfringens* neuraminidase per 1.9 cm^2^ well surface area. *Statistically significant difference (*P*≤0.02) between R6 and both R6Δ*nanA* and R6Δ*nanA*Δ*bgaA*. **Statistically significant difference between R6Δ*nanA* and R6Δ*nanA*+N (*P*≤0.02). (b) Reconstitution of *bgaA* restores adherence. All D562 cells were pretreated for 30 min with 0.01 U purified *C. perfringens* neuraminidase per 1.9 cm^2^ well surface area. *Statistically significant difference (*P*≤0.0001) between R6 and R6Δ*bgaA*. (c) Purified neuraminidase and rBgaA do not complement *bgaA* mutants. All D562 cells were pretreated for 30 min with 0.01 U purified *C. perfringens* neuraminidase per 1.9 cm^2^ well surface area. Where indicated (+B), neuraminidase treatment was followed by pretreatment with rBgaA at 4.33 pmol per 1.9 cm^2^ well surface area for 30 min. *Statistically significant difference (*P*≤0.05) between R6 and R6+B, R6Δ*bgaA* and R6Δ*nanA*Δ*bgaA*.

Previous data demonstrated that both *nanA* and *bgaA* mutants in R6 were reduced in adherence to the human upper airway epithelial cell line D562 (King *et al.*, 2006). Adherence of the *nanA* mutant (R6Δ*nanA*) could be complemented by addition of purified neuraminidase (King *et al.*, 2006). Data demonstrating that the double mutant (R6Δ*nanA*Δ*bgaA*) could not be complemented, even partially, by pretreatment with purified neuraminidase (Fig. 1a) suggest that both NanA and BgaA contribute to the same mechanism of adherence. Genetic reconstitution of R6*bgaA* (R6Δ*bgaA/bgaA*+) resulted in adherence not statistically significantly different from that of R6, confirming that mutation of *bgaA* was responsible for the reduction in adherence observed (Fig. 1b).

Complementation of the *nanA* mutant with purified enzyme suggests that the enzyme reveals a receptor for adherence. As NanA and BgaA act sequentially to deglycosylate host glycans, we initially proposed that β-galactosidase activity associated with BgaA acts following desialylation to expose surface receptors on host cells (King *et al.*, 2006). To test this hypothesis, we pretreated epithelial cells with neuraminidase and rBgaA. If BgaA was exposing a receptor for adherence, then pretreatment of epithelial cells would be expected to complement the *bgaA* mutant phenotype; however, neither R6Δ*bgaA* nor R6Δ*nanA*Δ*bgaA* was complemented (Fig. 1c and data not shown). Furthermore, rBgaA reduced adherence of the parental strain to a level not statistically significantly different from that of the BgaA mutant (R6Δ*bgaA*), even though we would have expected no change or perhaps an increase in adherence. Together, these data suggest an alternative role for BgaA in adherence.

### The role of BgaA in adherence is independent of its enzymic activity

**Fig. 2.**
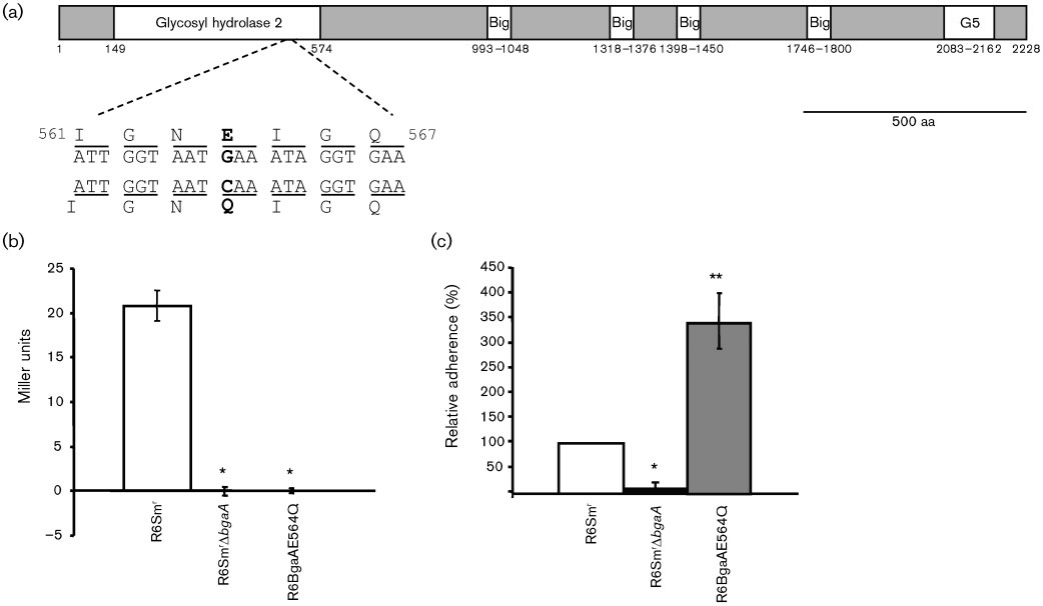
The role of BgaA in adherence is independent of its β-galactosidase activity. (a) Schematic representation of the published sequence of BgaA. Numbering refers to the amino acid locations in the published sequence, starting at the first putative methionine within the R6 *bgaA* published sequence (GenBank accession no. NC_003098; Hoskins *et al.*, 2001) and locations of conserved domains indicated: glycosyl hydrolase family 2 domain, bacterial Ig-like domains (Big) and the G5 domain. Nucleotide and amino acid sequences are given for regions flanking the site-directed mutation introduced to the BgaA active site. The mutated nucleotide and the resulting amino acid change are shown in bold beneath the parent sequence. (b) R6BgaAE564Q lacks detectable β-galactosidase activity. Aliquots of cultures, grown to an OD_600_ of 0.6, were lysed with toluene and activity was measured using the colorimetric substrate ONPG. Values are means±sd of three independent experiments. (c) R6BgaAE564Q is not significantly reduced in adherence compared with the parental strain R6Sm^r^. Bacterial strains were grown to an OD_600_ of 0.6 and their relative adherence to D562 cells was determined. Adherence is expressed in comparison to the percentage adherence of parental strain R6 over a 60 min incubation period under the same conditions. Values are means±sd of three independent experiments. *Statistically significant difference (*P*≤0.01) between R6Sm^r^ and R6Sm^r^Δ*bgaA* (b, c) and R6BgaAE564Q (b). **Statistically significant difference (*P*≤0.01) between R6BgaAE564Q and both R6Sm^r^ and R6Sm^r^Δ*bgaA*.

To start elucidating the role of BgaA in adherence, we investigated whether its β-galactosidase activity was required for efficient bacterial adherence. Enzymic activity is predicted to be encoded within the N-terminal domain, between aa 132 and 966, which shares significant sequence similarity with other sequenced β-galactosidases (Fig. 2a). Previous work in *E. coli* demonstrated that mutagenesis of the conserved glutamic acid residue 461 resulted in a 1000–7000-fold reduction in activity relative to the wild-type and a reduction in substrate binding (Bader *et al.*, 1988; Cupples & Miller, 1988; Cupples *et al.*, 1990; Gebler *et al.*, 1992; Richard *et al.*, 1996). Sequence analysis revealed a corresponding glutamic acid residue in *S. pneumoniae*, glutamic acid-564, and site-directed mutagenesis of this residue to glutamine (Fig. 2a) resulted in a strain, R6BgaAE564Q, that lacked all detectable β-galactosidase activity (Fig. 2b). Adherence of R6BgaAE564Q to D562 cells was significantly higher than that of R6Sm^r^Δ*bgaA* (*P*≤0.01; Fig. 2c). These data demonstrate that the role of BgaA in adherence is independent of β-galactosidase activity.

### BgaA blocks pneumococcal binding to epithelial cells

Pretreatment of epithelial cells with exogenous β-galactosidase reduced adherence of the R6 parental strain to a level not statistically significantly different from that of R6Δ*bgaA* (Fig. 1a). This reduction in adherence could be explained by the binding of rBgaA to bacterial receptors exposed by neuraminidase on the epithelial-cell surface, thereby blocking the ability of native BgaA on the bacterial-cell surface to mediate adherence. One interpretation of these data is that BgaA is acting as an adhesin.

**Fig. 3.**
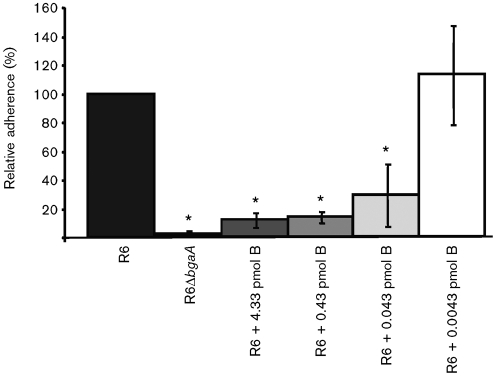
rBgaA competitively inhibits adherence of R6 to human epithelial cells in culture. R6 and R6Δ*bgaA* were grown to an OD_600_ of 0.6 and their relative adherence to D562 cells was determined. All D562 cells were pretreated for 30 min with 0.01 U purified *C. perfringens* neuraminidase per 1.9 cm^2^ well surface area. Indicated amounts of rBgaA per 1.9 cm^2^ well surface area were added to the bacterial inoculum (+B). Adherence is expressed in comparison to the percentage adherence of parental strain R6 over a 60 min incubation period under the same conditions. Values are means±sd of three independent experiments. *Statistically significant difference (*P*≤0.05) between R6, and R6+B and R6Δ*bgaA*.

To investigate this possibility, a competition assay was performed: a range of rBgaA concentrations was added to neuraminidase-treated epithelial cells at the same time as the R6 parental strain. Adherence was blocked in a dose-dependent manner and by as little as 0.043 pmol rBgaA per 1.9 cm^2^ well surface area (Fig. 3). This amount of rBgaA possesses β-galactosidase activity in tissue-culture medium not statistically significantly different from that produced by the number of bacteria added to our adherence assays and suggests a physiological relevance to these experiments. We confirmed that rBgaA had no toxic effect on *S. pneumoniae* or the epithelial cells by enumerating non-adherent bacteria and assessing the viability of monolayers (data not shown).

### BgaA binds directly to human epithelial cells

**Fig. 4.**
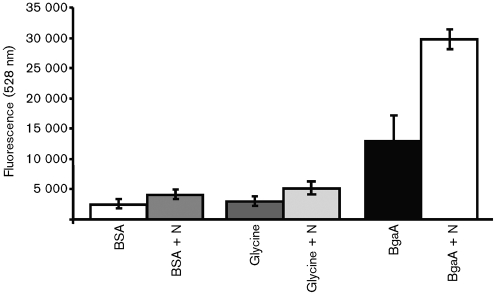
Relative binding of FluoSpheres (Invitrogen) coated with rBgaA, BSA or glycine to human epithelial cells in culture. Where indicated, D562 cells were pretreated for 30 min with 0.01 U purified *C. perfringens* neuraminidase (+N) per 1.9 cm^2^ well surface area. One hundred microlitres of a 1/1000 dilution of beads was added to each well and plates were incubated at 37 °C for 2 h. Plates were washed and dried, and binding was assessed by measuring fluorescence (528 nm). Shown is a representative of three independent experiments. Values are means±sd of three wells.

To further minimize the variables in the study of bacterial adherence, we compared the binding of FluoSpheres (Invitrogen) coated with rBgaA, glycine or BSA to untreated or neuraminidase-treated epithelial cells (Fig. 4). The adherence of BgaA-coated beads to neuraminidase-treated epithelial cells was at least 47.7 % higher than that of beads coated with BSA or glycine, thereby demonstrating that BgaA binds directly to human epithelial cells. In addition, treatment of the epithelial cells with neuraminidase increased the binding of BgaA-coated beads by at least 48.3 %, further supporting a role for pneumococcal neuraminidase in exposing a cell-surface receptor that mediates adherence. Neuraminidase treatment of epithelial cells also led to a small increase in adherence of all FluoSpheres, possibly due to the reduction of negatively charged sialic acid on the cell surface.

### Initial characterization of the BgaA receptor

Several pneumococcal adherence mechanisms have been elucidated. As R6 does not encode the adhesive pili, these do not contribute to adherence of this strain (Bagnoli *et al.*, 2008; Hoskins *et al.*, 2001). In addition, the glycosidase-dependent mechanism of adherence was shown to be independent of both the binding of phosphorylcholine on the bacterial cell to the PAFR, and the binding of CbpA to the polymeric immunoglobulin (Ig) receptor, as neither the R6Δ*cbpA* strain nor treatment with PAFR inhibitor reduced adherence in our models (data not shown) (Cundell *et al.*, 1996; Zhang *et al.*, 2000).

**Fig. 5.**
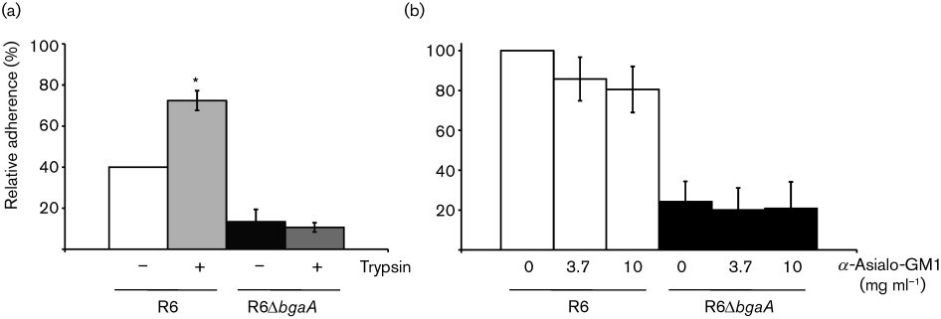
Studies to determine the nature of the epithelial-cell receptor for BgaA. R6 and R6 exoglycosidase mutants were grown to an OD_600_ of 0.6 and their relative adherence to D562 cells was determined. Adherence is expressed in comparison to the percentage adherence of parental strain R6 over a 60 min incubation period under the same conditions. Values are means±sd of three independent experiments. (a) Trypsin treatment does not alter the adherence of R6 to airway epithelial cells. Where indicated (+), epithelial cells were pretreated with 200 µl 0.0025 % trypsin. *Statistically significant difference (*P*≤0.01) between R6 and R6+trypsin. (b) Addition of anti-asialo-GM1 does not reduce R6 adherence. All cells were pretreated for 30 min with 0.01 U purified *C. perfringens* neuraminidase per 1.9 cm^2^ well surface area. The concentrations of anti-asialo-GM1 indicated were added to the inoculum.

To determine whether BgaA is binding to a proteinaceous receptor, epithelial cells were pretreated with protease. Pretreatment of cells with trypsin did not reduce the adherence of R6; in fact, a statistically significant increase in adherence was observed (*P*≤0.01; Fig. 5a). These data suggest that R6 is binding to a non-proteinaceous receptor that is exposed more efficiently on the epithelial-cell surface following the removal of proteins. Furthermore, in this model system, the residual adherence of the BgaA mutant does not appear to be due to a protein receptor, as trypsin pretreatment did not alter adherence of R6Δ*bgaA* significantly. Pretreatment of cells with proteinase K (1 mg ml^−1^) showed similar results (data not shown). These data suggest that the receptor is likely to be a glycan in the context of a glycosphingolipid.

*S. pneumoniae* has previously been proposed to bind to the glycosphingolipid asialo-GM1 (Krivan *et al.*, 1988). However, whether *S. pneumoniae* binds to this structure on epithelial cells or what may serve as the bacterial adhesin has not been determined. To determine whether R6 binds to asialo-GM1, adherence assays were performed following the pre-incubation of neuraminidase-treated epithelial cells with anti-asialo-GM1 (Fig. 5b). Addition of this antibody was shown previously to reduce adherence of bacteria that utilize asialo-GM1 as an adhesin (de Bentzmann *et al.*, 1996). However, no statistically significant reduction in adherence of R6 or R6Δ*bgaA* was observed. These data suggest that R6 does not bind to asialo-GM1 under these assay conditions. Furthermore, adherence to epithelial cells was not reduced when bacteria were pre-incubated with asialo-GM1 (data not shown). Together, these data suggest that *S. pneumoniae* binds to a glycosphingolipid, but not asialo-GM1.

### BgaA contributes to adherence of encapsulated pneumococci to epithelial cells of different origins

**Fig. 6.**
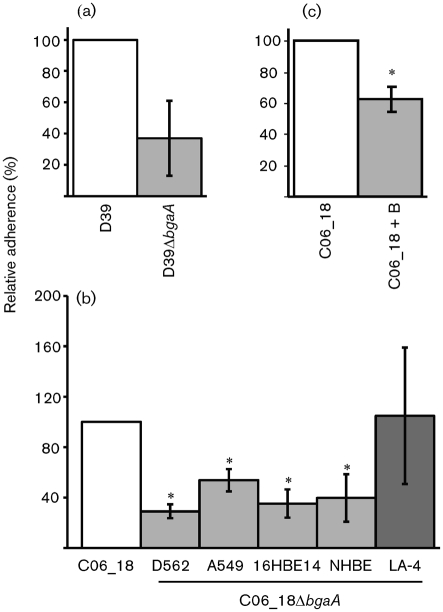
BgaA contributes to the adherence of encapsulated pneumococci to human epithelial cells. Bacterial strains were grown to an OD_600_ of 0.6. Adherence is expressed relative to the adherence of the parental strain over a 60 min incubation period under the same conditions. Values are means±sd of three independent experiments. (a) Relative adherence of D39 and D39Δ*bgaA* to D562 epithelial cells in culture. *Statistically significant difference (*P*≤0.01) between the relative adherence of D39Δ*bgaA* and its parental strain. (b) Relative adherence of C06_18Δ*bgaA* to epithelial cells in culture. The relative adherence of C06_18Δ*bgaA* to D562, A549, HBE, NHBE and LA-4 epithelial cells was determined. *Statistically significant difference (*P*≤0.01) between the relative adherence of C06_18Δ*bgaA* and its parental strain. (c) rBgaA significantly reduces adherence of an encapsulated recent clinical isolate. Where indicated (+B), 4.33 pmol rBgaA per 1.9 cm^2^ well surface area was added to the bacterial inoculum. *Statistically significant difference (*P*≤0.002) between the relative adherence of C06_18 in the presence of rBgaA.

R6 is an unencapsulated strain of *S. pneumoniae*. Whilst there is evidence that *S. pneumoniae* expresses lower levels, or is devoid of, capsule polysaccharide during colonization of the upper airway, expression of the capsular polysaccharide is required for the ability of the bacterium to cause invasive disease (Hammerschmidt *et al.*, 2005; Kim *et al.*, 1999; Watson & Musher, 1990). We therefore demonstrated that a mutant in D39 (D39Δ*bgaA*), the encapsulated progenitor strain of R6, was also statistically significantly reduced in adherence to D562 cells compared with the parental strain (*P*≤0.01; Fig. 6a).

We also wanted to investigate the contribution of BgaA to adherence of more recent isolates; therefore, we generated BgaA mutants in C06_18, 384 and TIGR4. Mutation of *bgaA* statistically significantly reduced adherence of one of the three strains: C06_18, a low-passage serotype 22F strain isolated from blood (*P*≤0.01; Fig. 6b and data not shown). A second independent mutant generated in this strain background showed the same phenotype, thereby confirming that mutation of *bgaA* was responsible for the observed phenotype. To further support the hypothesis that BgaA contributes to adherence of C06_18, we confirmed that addition of rBgaA statistically significantly reduced adherence of this strain (*P*≤0.002). Together, these data suggest that BgaA acts as an adhesin for at least some clinical isolates (Fig. 6c).

As D562 cells represent only a small fraction of the human airway at best, similar experiments were performed using a human lung carcinoma cell line, A549, and an immortalized human bronchial epithelial cell line, 16HBE14 (Fig. 6b). The level of adherence of C06_18 to each of these cell lines was different, perhaps suggesting the differential distribution of receptors on the epithelial-cell surface (mean adherence of C06_18 to D562, 2.0±1.1×10^4^ per well; to 16HBE14, 4.9±4.5×10^4^ per well; and to A549, 1.2±2.8×10^4^ per well). However, when compared with the parental strain, C06_18Δ*bgaA* showed a statistically significant reduction in adherence to all three human cell lines (*P*≤0.01).

The BgaA mutant (C06_18Δ*bgaA*) showed no reduction in ability to colonize mice compared with the parental strain [C06_18, 4.75±0.67×10^4^; C06_18Δ*bgaA*, 5.64±1.65×10^4^ (ml lavage fluid)^−1^]. This suggests that the receptor may be human-specific. The demonstration that C06_18Δ*bgaA* was not reduced in adherence to a neuraminidase-treated mouse airway epithelial cell line, LA-4 (Fig. 6b; C06_18, 1.7±1.7×10^4^; C06_18Δ*bgaA*, 2.1±2.2×10^4^ per well), further supports this hypothesis (Stoner *et al.*, 1975).

As immortalization of cells can lead to changes in expression of surface molecules, including receptors (Edwards *et al.*, 2001), we also demonstrated that C06_18Δ*bgaA* was statistically significantly reduced (60 %) in adherence to primary normal human bronchial epithelial cells (*P*≤0.01; mean adherence of C06_18 to NHBEs, 5.65±8.5×10^4^ per well) (Lonza) (Fig. 6b). In addition to C06_18, R6Δ*bgaA* was also shown to have statistically significantly reduced adherence to immortalized and primary human epithelial cells (*P*≤0.01; data not shown). Together, these data suggest that BgaA contributes to adherence of recent clinical isolates to human airway epithelial cells of different origins.

## Discussion

Carbohydrate modification plays an essential role in pneumococcal pathogenesis; carbohydrates are required for growth of the bacteria and compose the pneumococcal capsule, which is essential for disease. The finding that BgaA probably acts as an adhesin supports the hypothesis that pneumococcal glycosidases contribute to colonization independently of their enzymic activity. This hypothesis is also supported by the recent finding that the lectin-like domain of NanA promotes pneumococcal adherence and invasion of human brain microvascular endothelial cells (Uchiyama *et al.*, 2009). Our current study indicates that NanA reveals a receptor on airway epithelial cells. Together, these findings suggest distinct roles for NanA in adherence to different cell types, probably due to the differential distribution of receptors.

Pneumococcal adherence appears to be complex. Several mechanisms of pneumococcal adherence have been identified previously (Cundell *et al.*, 1995b; Hammerschmidt, 2006; Zhang *et al.*, 2000). Given the importance of bacterial adherence to colonization and different stages of disease, it is not surprising that *S. pneumoniae* possesses multiple adherence mechanisms. Adding another layer of complexity is the fact that the expression of some pneumococcal adhesins differs between strains (Bagnoli *et al.*, 2008; Moschioni *et al.*, 2008; Paterson & Mitchell, 2006). Furthermore, it is likely that different host cells vary in their expression of receptors for adherence. Thus, we can predict that the relative contribution of different mechanisms to adherence is probably dependent on both the host-cell type and the pneumococcal strain. We demonstrated that *bgaA* contributed significantly to adherence of R6, D39 and C06_18. The residual adherence observed is probably due to expression of other adherence mechanisms. We were unable to demonstrate a phenotype for two further strains, TIGR4 and 384. It is unknown whether these strains express other adherence mechanisms that compensate completely for the absence of BgaA or whether this protein does not contribute to adherence of these strains. Our data allow us to conclude that BgaA contributes to adherence of at least some pneumococcal strains to all airway epithelial cells tested, including primary NHBE cells.

Our experiments demonstrated that rBgaA inhibited pneumococcal adherence and bound directly to epithelial cells, both key characteristics of adhesins. Analysis of the BgaA sequence reveals features supporting the hypothesis that this protein may have functions besides that of a β-galactosidase (Fig. 2a). BgaA is approximately twice the size of the majority of other predicted β-galactosidases. The N-terminal region of the protein (aa 110–1136) shares significant sequence similarity with other β-galactosidases, but the C-terminal half of the protein, with the exception of the surface-localization marker, has no known function and lacks sequence similarity to most predicted β-galactosidases. The uncharacterized, predicted β-galactosidases from some other streptococcal species, including *Streptococcus oralis* (ATCC 35037^T^; AEDW01000000), *Streptococcus mitis* (ATCC 6249; AEEN01000000), *Streptococcus sanguinis* (ATCC 49296; AEPO01000000) and *Streptococcus gordonii* (Vickerman *et al.*, 2007), are approximately the same length as BgaA and share high levels of amino acid identity over 2166 aa. Whether BgaA is a member of a novel family of β-galactosidase adhesins is unknown.

The region of BgaA that mediates adherence is currently unknown. The C-terminal region may confer the Galβ1,4GlcNAc enzyme specificity or perform a second independent function, for example mediating adherence. Homology searches using the C terminus of BgaA did not reveal a function for this region; however, it does contain an 80 aa partial G5 domain (aa 2083–2162). G5 domains are present in many enzymes that bind GlcNAc and have been proposed to contribute to this binding (Bateman *et al.*, 2005). This G5 domain may contribute to BgaA-mediated adherence, either directly by enabling binding to surface-associated GlcNAc or indirectly by contributing to the restricted cleavage specificity of BgaA. This may in turn enable BgaA to adhere to glycan substrates that it is unable to cleave. G5 domains present in staphylococcal surface proteins have also been proposed to mediate intercellular adhesion, although a single repeat was insufficient for this activity (Conrady *et al.*, 2008). Also, within the C-terminal region are several bacterial Ig folds (pfam07532), which are often identified in both glycosidases and other surface proteins (Marchler-Bauer *et al.*, 2007). The role of the G5 domain and Ig folds will be the focus of further research.

Generation of the active-site mutant R6BgaAE564Q demonstrated that residue 564 is essential for enzymic activity and confirmed that β-galactosidase activity is not required for BgaA to mediate pneumococcal adherence. Mutation of the corresponding residue in *E. coli* was reported to reduce substrate binding (Cupples *et al.*, 1990); however, it is unknown whether this is true in *S. pneumoniae*. R6BgaAE564Q adhered to epithelial cells at a significantly higher level than the parental strain, raising several possibilities for the increased adherence. If the active site is responsible for adherence and the glutamine substitution at aa 564 does not reduce substrate binding, Galβ1,4GlcNAc could act as an additional receptor on the epithelial-cell surface. Alternatively, if the region mediating adherence is distinct from the active site, it is possible that the substrate is still bound efficiently and that the inability to cleave this linkage may provide more time for increased interaction between the adhesin and receptor. A third possibility is that substrate binding is reduced, and that BgaA which is not interacting with the substrate has increased ability to bind to the receptor.

The receptor for BgaA on the epithelial surface is currently unknown. Previous studies have reported that *S. pneumoniae* adheres to a number of different host-cell glycoconjugates, including GalNAcβ1,4Gal found in ganglioside GM1 (Krivan *et al.*, 1988). However, our studies suggest that BgaA is binding to the glycan portion of a glycosphingolipid, but not GM1. It has previously been proposed that the initial adherence of *S. pneumoniae* to resting epithelial cells is mediated by a glycoconjugate receptor (Cundell *et al.*, 1995a; Hammerschmidt, 2006); therefore, NanA and BgaA may contribute to the initial adherence of *S. pneumoniae* to human epithelia. Mutation of *bgaA* was shown previously to have a small but significant effect in a model of pneumonia, but the role of this protein in colonization was not examined (Robertson *et al.*, 2002). We observed no reduction in the ability of a *bgaA* mutant to colonize the oro-nasopharynx or to bind to mouse airway epithelial cells, suggesting that the BgaA receptor is human-specific.

Collectively, these data support the hypothesis that pneumococcal glycosidases have functions independent of their enzymic activity. Glycosidases are probably upregulated during colonization, as the concentration of free carbohydrates in the nasopharynx is normally low and the bacteria require carbon for growth. Additional functions for these proteins may efficiently co-ordinate growth and other processes, including adherence, that are required to establish and maintain colonization.
